# Effects of Dietary Protein Intake Levels on Peripheral Circadian Rhythm in Mice

**DOI:** 10.3390/ijms25137373

**Published:** 2024-07-05

**Authors:** Yerim Han, Jinyoung Shon, So Young Kwon, Yoon Jung Park

**Affiliations:** 1 Department of Nutritional Science and Food Management, Ewha Womans University, Seoul 03760, Republic of Korea; hanyelim97@naver.com (Y.H.); shon.jinyoung.layla@gmail.com (J.S.); kwongood98@naver.com (S.Y.K.); 2 Graduate Program in System Health Science and Engineering, Ewha Womans University, Seoul 03760, Republic of Korea

**Keywords:** diets, nutrients, low-protein diet, circadian clock, circadian rhythm, endoplasmic reticulum (ER) stress

## Abstract

The regulation of the circadian clock plays an important role in influencing physiological conditions. While it is reported that the timing and quantity of energy intake impact circadian regulation, the underlying mechanisms remain unclear. This study investigated the impact of dietary protein intake on peripheral clocks. Firstly, transcriptomic analysis was conducted to investigate molecular targets of low-protein intake. Secondly, *mPer2::Luc* knock-in mice, fed with either a low-protein, normal, or high-protein diet for 6 weeks, were analyzed for the oscillation of PER2 expression in peripheral tissues and for the expression profiles of circadian and metabolic genes. Lastly, the candidate pathway identified by the in vivo analysis was validated using AML12 cells. As a result, using transcriptomic analysis, we found that the low-protein diet hardly altered the circadian rhythm in the central clock. In animal experiments, expression levels and period lengths of PER2 were different in peripheral tissues depending on dietary protein intake; moreover, mRNA levels of clock-controlled genes and endoplasmic reticulum (ER) stress genes were affected by dietary protein intake. Induction of ER stress in AML12 cells caused an increased amplitude of *Clock* and *Bmal1* and an advanced peak phase of *Per2*. This result shows that the intake of different dietary protein ratios causes an alteration of the circadian rhythm, especially in the peripheral clock of mice. Dietary protein intake modifies the oscillation of ER stress genes, which may play key roles in the regulation of the circadian clock.

## 1. Introduction

Circadian rhythm plays a critical role in the regulation of metabolic function [1,2]. Abnormal physiological conditions, such as obesity or diabetes, are associated with dysregulation of the circadian clock. The diurnal amplitude of circadian gene expression was reduced in the adipose tissue of individuals with type 2 diabetes [3]. In addition, the circadian clock is involved in the aging process, which leads to substantial changes in the circadian regulation of metabolic clocks in a tissue-specific manner [4]. Aging-induced circadian disruption in the liver is mainly linked to the alteration of NAD^+^ metabolism and protein acetylation [4,5]. Therefore, the rhythmicity of the circadian clock and its targets can be useful biomarkers for overall health status and lifespan.

Dietary patterns, such as the amount of intake and eating time, influence peripheral clocks by regulating the oscillation of circadian clock genes and, subsequently, clock-controlled metabolic genes [6,7,8,9,10,11,12,13,14]. For example, 30% calorie restriction not only changed expression patterns of the period (*Per1* and *Per2*), aryl hydrocarbon receptor nuclear translocator-like (*Arntl*, also known as *Bmal1*), and cryptochrome 2 (*Cry2*) genes in the mouse liver but also altered their amplitude in a BMAL1-dependent manner [6]. Time-restricted feeding for 12 weeks resulted in the refined hepatic oscillation of circadian genes, such as *Per2*, *Bmal1*, *Rev-erbα*, and cryptochrome 1 (*Cry1*), with increased amplitude in mice [10,15]. In addition, diets with varying compositions, such as a high-fat diet (HFD), alter peripheral clocks, particularly in the liver [16,17]. The evidence suggests that different dietary macronutrient compositions might undermine or restore circadian synchrony.

The effects of macronutrient compositions on metabolism have been extensively studied, while studies on their effects on circadian regulation are limited. A key challenge is to reconcile the effects of dietary protein intake on health and longevity [18]. A high-protein diet (HPD) has received growing attention as a type of health-promoting diet that enriches muscle mass, lowers appetite and weight gain, and decreases blood pressure and serum lipid levels in humans, especially among the elderly [19,20]. Protein-fortified food items and supplements are widely popular in the markets of developed countries [21]. Moreover, some health professionals have recommended the consumption of an HPD to prevent chronic diseases and promote metabolic regulation. However, the validity and safety of an HPD have not been fully identified [22]. Intriguingly, recent evidence has suggested that the low-protein diet (LPD) might be beneficial for health improvement and longevity in people with certain physiological conditions [18,23,24]. Simpson et al. [25] developed a state-space model of nutrition (the Geometric Framework), showing that metabolic health and longevity were not gained in HPD-fed mice but rather with low-protein/high-carbohydrate diets [18]. Furthermore, a human study showed that an LPD decreased diabetes-related mortality in subjects over 50 years of age but elevated all-cause mortality in subjects over 65 years of age [24]. The varying outcomes from different levels of dietary protein underscore the importance of comprehensibly grasping the overall physiological response, like the circadian clock, to protein levels.

In this study, we investigated how dietary protein intake levels affect the circadian clock and identified the possible underlying mechanisms. The study aimed to evaluate the impact of dietary protein intake on the regulation of the circadian and peripheral clocks in mice, particularly in the liver. Additionally, the study aimed to identify a potential mechanism through which protein intake may affect the synchrony of the circadian clock, using both in vivo and in vitro models.

## 2. Results

### 2.1. Hypothalamic Expression of Circadian Clock Genes Was Not Altered by a Low-Protein Diet

**Figure 1:**
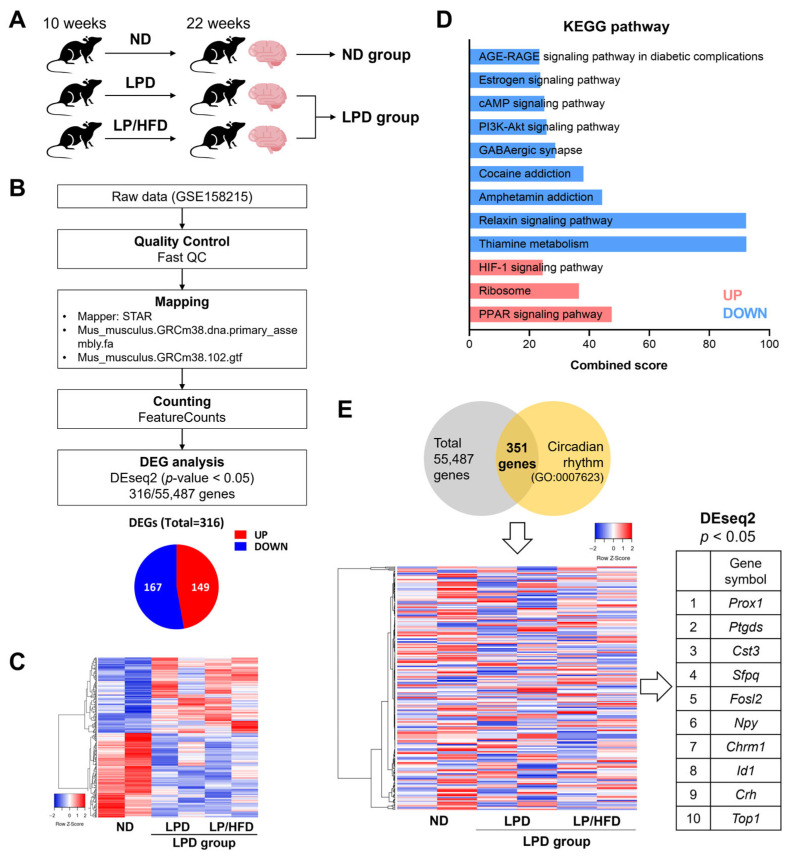
Analysis of the hypothalamic transcriptome in mice using publicly available RNA-sequencing data. (**A**) Experimental design of GSE158215. For secondary analysis, the low-protein diet (LPD) and low-protein/high-fat diet (LP/HFD) groups were merged into the LPD group. (**B**) Analytical procedure for filtering differentially expressed genes (DEGs) from RNA-sequencing raw data of GSE158215. (**C**) Heatmap presenting expression of DEGs in the LPD group versus the normal diet (ND) group. Each column represents a sample, and each row represents a gene. The red and blue colors indicate upregulated and downregulated genes, respectively. (**D**) Results of the Kyoto Encyclopedia of Genes and Genomes (KEGG) pathway analysis. Upregulated and downregulated pathways with *p*-values < 0.01 and combined scores are shown. (**E**) Heatmap presenting expression of genes involved in the gene ontology term “Circadian rhythm” (GO:0007623) among a total of 55,487 genes and a list of DEGs. The red and blue colors indicate upregulated and downregulated genes, respectively.

First, through the secondary analysis of publicly available transcriptome data (GSE158215) [26], we tested whether dietary protein intake level changes the circadian regulation of the central clock in the hypothalamus. The data we used were based on RNA-sequencing analysis of the hypothalamus in mice fed with three different diets for 12 weeks: normal diet (ND) with 20% protein and 20% fat, LPD with 5% protein and 20% fat, and low-protein/high-fat diet (LP/HFD) with 5% protein and 60% fat. To understand the impact of an LPD regardless of fat content, differentially expressed genes (DEGs) were identified through a comparison between the LPD groups (LPD and LP/HFD) and the ND group at *p* < 0.05 (Figure 1A–C). The pathway analysis based on the DEGs revealed that the peroxisome proliferator-activated receptor (PPAR) signaling pathway and the hypoxia-inducible factor-1 (HIF-1) signaling pathway were elevated in the LPD group, and thiamine metabolism and the relaxin signaling pathway were downregulated in the LPD group (Figure 1D). Further analysis focused on 351 genes belonging to gene ontology (GO) terms associated with the circadian rhythm (GO:0007623) and revealed minimal changes in their expression (Figure 1E). Only 10 of these genes, which are not core clock genes, were differentially expressed, and these 10 genes were all marginally significant (Figure 1E). The results demonstrated that the central hypothalamic circadian clock might not be a direct target of low consumption of dietary protein.

### 2.2. Low-Protein Diet-Influenced Bodyweight, Food Intake, and Glucose Tolerance

**Figure 2:**
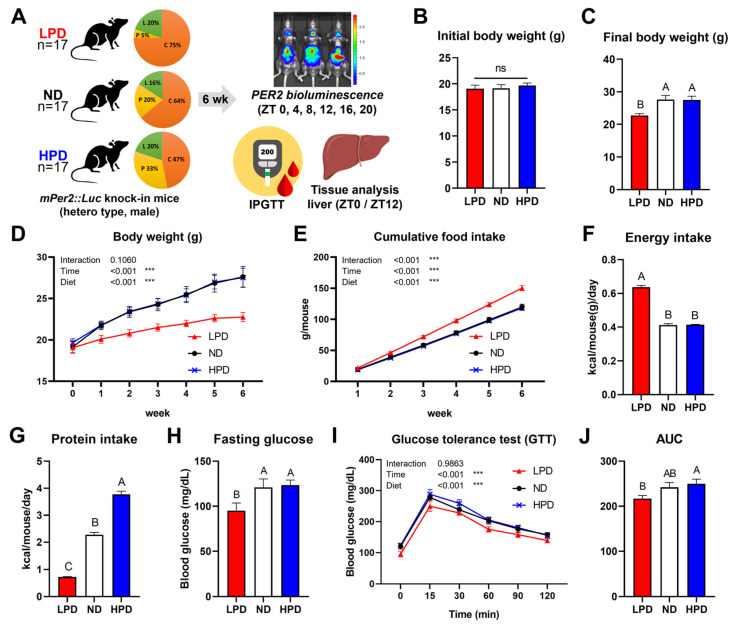
Effects of a low-protein diet (LPD) on body weight (BW), food intake, and glucose tolerance. (**A**) Schematic overview of in vivo experiments. (**B**) Initial BW. (**C**) Final BW after 6 weeks of dietary intervention. (**D**) BW change in mice during dietary intervention. (**E**) Cumulative average food intake of individual mice. (**F**) Average daily energy intake normalized to BW. (**G**) Average daily energy intake from protein. (**H**) Fasting blood glucose levels. (**I**) Blood glucose levels at 0, 15, 30, 60, and 120 min after glucose injection. (**J**) Area under the curves (AUCs) calculated from (**I**). (**B**–**G**): *n* = 12 biological replicates; (**H**–**J**): *n* = 8 biological replicates. The data are presented as means ± standard errors. One-way ANOVA and Duncan’s post hoc test were used for statistical analysis and indicated by different letters (ns, not significant). *p* < 0.05 was considered statistically significant. Two-way ANOVA and Duncan’s post hoc test were used to determine statistically significant effects of diet, time, and their interaction (*** *p* < 0.001). GTT, glucose tolerance test; ZT, zeitgeber time.

We hypothesized that the level of dietary protein intake might influence the peripheral clocks. To investigate this hypothesis, *mPer2::Luc* knock-in mice were used. The mice were fed with one of three diets: LPD, ND, or HPD, which differed in protein content (5%, 20%, and 33% protein, respectively) (Figure 2A). After the 6-week ad libitum dietary intervention, we monitored in vivo PER2 expression and assessed body weight (BW), food intake, and glucose tolerance (Figure 2A). Initial BW was not significantly different among the three groups, but after 1 week of dietary intervention, the BW of the LPD group was significantly lower compared to the other groups (Figure 2B–D). Among the three groups, food intake and calorie intake were highest in the LPD group (Figure 2E,F). However, the calorie intake from protein was lowest in the LPD group and highest in the HPD group (Figure 2G). These results show that the increased food intake in the LPD group could not compensate for a protein intake deficiency. The fasting glucose level of the LPD group was lower than those of the other groups (Figure 2H), and the blood glucose levels during the glucose tolerance test (GTT) were different depending on the diet (Figure 2I). The area under the curve (AUC) for blood glucose was higher in HPD-fed mice than in LPD-fed ones, but neither of these groups was significantly different from the ND group (Figure 2J).

### 2.3. The Level of Dietary Protein Intake Altered Peripheral Circadian Clocks

**Figure 3:**
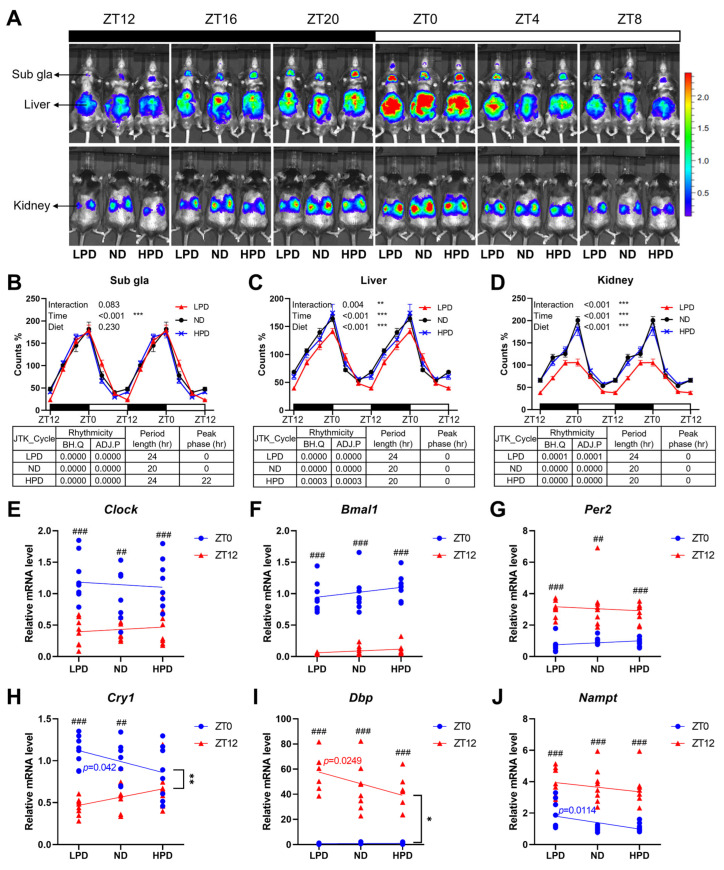
Results of in vivo monitoring of PER2 expression and hepatic expression of circadian genes in *mPer2::Luc* knock-in mice fed with diets consisting of different protein ratios. (**A**) Representative images from in vivo monitoring of PER2 expression in *mPer2::Luc* knock-in mice fed with diets consisting of different protein ratios at each timepoint with 4 h intervals. Bioluminescence image obtained from the ventral-up position for the submandibular gland (sub gla) and the liver and the dorsal-up position for the kidney. (**B**–**D**) In vivo bioluminescence levels from the sub gla (**B**), liver (**C**), and kidney (**D**). The average count percentages were double-plotted according to time. *n* = 4 biological replicates. The data are presented as means ± standard errors. BH.Q is the Benjamini–Hochberg *q*-value, and ADJ.P is the Bonferroni adjusted *p*-value. Two-way ANOVA and Duncan’s post hoc test were used to determine statistically significant effects of diet, time, and their interaction (* *p* < 0.05, ** *p* < 0.01, *** *p* < 0.001). JTK_cycle analysis was performed to measure rhythmicity, period length, and peak phase. (**E**–**J**) Hepatic expression levels of circadian clock genes *Clock* (**E**), *Bmal1* (**F**), *Per2* (**G**), *Cry1* (**H**), *Dbp* (**I**), and *Nampt* (**J**) at zeitgeber time (ZT) 0 and ZT 12. The data were normalized to the expression of the *Tbp* gene. *n* = 8 biological replicates. The data are presented as means ± standard errors. Linear regression analysis was used to test the relationship between mRNA levels and protein intake levels at each ZT. If slopes were significantly non-zero, the *p*-value is presented with a blue or red color, respectively, for ZT 0 or ZT 12, indicating that mRNA levels differ depending on protein intake level. Statistical analysis for comparison between two slopes was conducted (* *p* < 0.05, ** *p* < 0.01, *** *p* < 0.001). Student *t*-tests were used to compare between ZT 0 and ZT 12 within each group (^##^ *p* < 0.01, ^###^ *p* < 0.001).

Every 4 h for 24 h, PER2 expression in the liver, kidney, and submandibular gland (sub gla) was measured using an in vivo imaging system (IVIS) (Figure 3A). In the sub gla, period lengths were longer in the HPD and LPD groups than in the ND group, and the peak phase of the HPD group was advanced (Figure 3B). In the liver and kidney, the period length of the LPD group was longer than those of the other groups (Figure 3C,D). Furthermore, bioluminescence levels differed depending on the diet, time, and diet × time interaction (Figure 3C,D). This result shows that dietary protein intake alters the oscillation of PER2 with tissue-specificity in peripheral clocks: sub gla, liver, and kidney.

To decipher the effects of dietary protein intake on the oscillation of circadian genes in a major metabolic center of the body, the liver was collected at zeitgeber time (ZT) 0 and ZT 12. As well as the expression of the clock circadian regulator (*Clock*), the *Bmal1* and *Per2* genes had no significant regression depending on protein intake, and oscillating changes between expression levels at ZT 0 and ZT 12 were retained with different diets (Figure 3E–G). The expression of D-box binding PAR BZIP transcription factor (*Dbp*) and nicotinamide phosphoribosyltransferase (*Nampt*), which are clock-controlled genes, showed day–night differences in all diet groups, which meant that the oscillation of expression was maintained (Figure 3I,J). However, the mRNA levels of *Cry1* in the HPD group had no significance between ZT 0 and ZT 12, which showed that the oscillation pattern of *Cry1* had changed (Figure 3H). The expression levels of *Cry1*, *Dbp*, and *Nampt* had a negative correlation with protein intake at ZT 12, ZT 12, and ZT 0, respectively (Figure 3H–J). Furthermore, the expression patterns of *Cry1* and *Dbp* were significantly different between ZT 0 and ZT 12 (Figure 3H,I). These showed that the oscillating patterns of clock-controlled genes were influenced by dietary protein intake.

### 2.4. The Level of Dietary Protein Intake Shifted the Expression and Oscillation of Metabolic Genes

**Figure 4:**
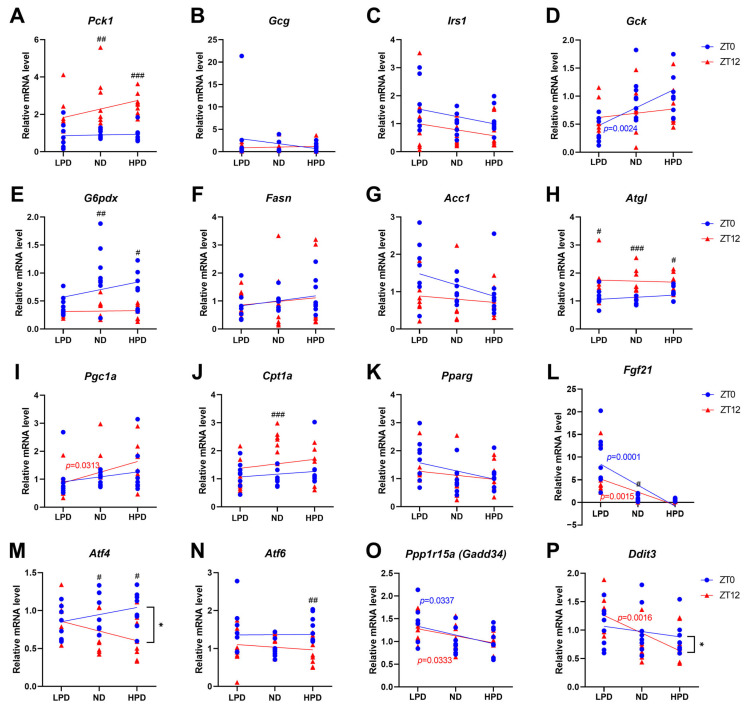
Effects of dietary protein intake on the expression pattern of metabolic genes. (**A**–**E**) Expression levels of genes regulating glucose metabolism: *Pck1* (**A**), *Gcg* (**B**), *Irs1* (**C**), *Gck* (**D**), and *G6pdx* (**E**). (**F**–**K**) Expression levels of genes regulating lipid metabolism: *Fasn* (**F**), *Acc1* (**G**), *Atgl* (**H**), *Pgc1a* (**I**), *Cpt1a* (**J**), and *Pparg* (**K**). (**L**) Expression levels of *Fgf21.* (**M**,**N**) Expression levels of genes related to an endoplasmic reticulum (ER) stress response: *Atf4* (**M**), *Atf6* (**N**), *Ppp1r15a* (**O**), and *Ddit3* (**P**). mRNA expression levels were measured by qRT-PCR analysis, and the data were normalized to the expression of the *Tbp* gene. *n*= 8 biological replicates per time. Linear regression analysis was used to test the relationship between mRNA levels and protein intake. If the slope was significantly non-zero, the *p*-value is presented with a blue or red color, respectively, for zeitgeber time (ZT) 0 or ZT 12, indicating that mRNA levels differ depending on protein intake level. Statistical analysis for comparison between two slopes was conducted (* *p* < 0.05). Student *t*-tests were used to compare between ZT 0 and ZT 12 within each group (^#^ *p* < 0.05, ^##^ *p* < 0.01, ^###^ *p* < 0.001).

We tested whether metabolic pathways were altered by dietary protein intake. Firstly, genes associated with glucose metabolism were analyzed because it is the main energy metabolism in mammals. Although the expression level of phosphoenolpyruvate carboxykinase 1 (*Pck1*) had no relation to protein intake, the change in expression levels at ZT 0 and ZT 12 was lost in the LPD group, indicating disruption of the circadian oscillation (Figure 4A). The expression levels and oscillation patterns of glucagon (*Gcg*) and insulin receptor substrate 1 (*Irs1*) were not changed by dietary protein intake (Figure 4B,C), but the expression level of glucokinase (*Gck*) at ZT 0 had a positive relationship with dietary protein intake (Figure 4D). The day–night difference in *Pck1* and glucose-6-phosphate 1-dehydrogenase (*G6pdx*) expression was attenuated in the LPD group (Figure 4E).

Next, the mRNA levels of genes associated with lipid metabolism were measured. The expression levels of fatty acid synthase (*Fasn*) and acetyl-CoA carboxylase 1 (*Acc1*), which are involved in the lipogenesis pathway, were not affected by dietary protein intake (Figure 4F,G). Only peroxisome proliferator-activated receptor gamma coactivator 1 alpha (*Pgc1a*) had a relationship with protein intake at ZT 12 (Figure 4I). Although the expression levels and patterns of adipose triglyceride lipase (*Atgl*), carnitine palmitoyltransferase 1A (*Cpt1a*), and peroxisome proliferator-activated receptor gamma (*Pparg*) had no relationship with the dietary intervention (Figure 4H,K), the day–night difference in *Cpt1a* expression disappeared in the HPD and LPD groups (Figure 4J). The disappearance of a significant difference in gene expression between day and night may mean that a low-protein intake disrupts circadian oscillations or changes the circadian phase (Figure 4A,E,J).

### 2.5. Endoplasmic Reticulum (ER) Stress Response Is a Potential Pathway Regulating Circadian Rhythm in Peripheral Tissues

To identify which pathway might be involved in the altered circadian regulation upon metabolic stress, we performed another secondary analysis using the microarray data (GSE52333) obtained in a study by Eckel-Mahan et al. (2013) [17] that characterized changes in the circadian liver transcriptome of mice fed with an ND or HFD for 6 weeks (Supplemental Figure S1A). With a *p*-value cut-off < 0.01, we selected 691 DEGs among a total of 27,359 genes for biological pathway analysis (Supplemental Figure S1B). In addition to fatty acid metabolism and lipid metabolism, the ER unfolded protein response (UPR) and response to ER stress were significantly altered in HFD-fed mice (Supplemental Figure S1C,D). In addition, among genes involved in GO:0007623, the expression of 39 circadian genes was influenced by HFD feeding, which can be evidence of a correlation between the peripheral clock and dietary stress from an HFD.

In a further investigation, the expression of fibroblast growth factor 21 (*Fgf21*), which has been considered mainly a starvation marker, was measured. The mRNA level of *Fgf21* was dramatically increased in the LPD group at ZT 0 and ZT 12 (Figure 4L). In a previous study, inducing ER stress raised the expression of FGF21 in vitro and in vivo through the eukaryotic translation initiation factor 2A (eIF2α)—activating transcription factor 4 (ATF4) pathway [27]. Thus, we investigated the changes in ER stress-response genes.

Through linear regression analysis, it was found that the expression levels of *Atf4* had no relationship with protein intake; however, the day–night difference in the expression of this gene was lost by LPD feeding (Figure 4M). Although the expression levels of activating transcription factor 6 (*Atf6*) had no significant tendency depending on dietary protein intake, the day–night difference in its expression was increased in the HPD group (Figure 4N). Furthermore, the lower the protein intake, the higher the expression of protein phosphatase 1 regulatory subunit 15A (*Ppp1r15a*) at ZT 0 and ZT 12, and there were no day–night differences in its expression among all groups (Figure 4O). The expression of DNA damage-inducible transcript 3 (*Ddit3*) at ZT 12 was significantly higher in the LPD group than in the other groups (Figure 4P). Although there was no difference in its expression between ZT 0 and ZT 12 in all groups, its tendency of expression at ZT 0 and ZT 12 in the LPD group was opposite to that in the other groups (Figure 4P). Overall, low intake of dietary protein increased the expression of ER stress genes in mice liver. These results show that diets composed of different proportions of protein influence parts of metabolic pathways, including the ER stress pathway, not only expression levels but also day–night differences indicating oscillation.

**Figure 5:**
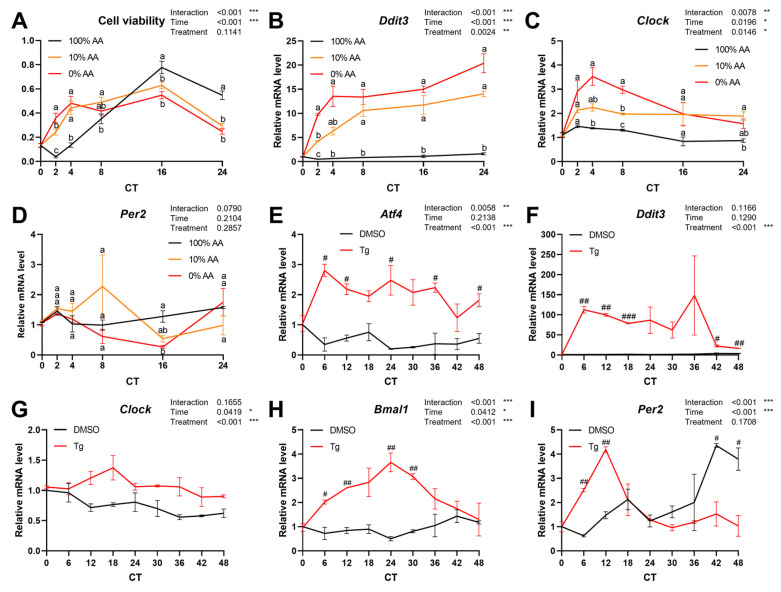
Investigation of the mechanism transmitting the signals from protein availability to the circadian clock in AML12. (**A**–**D**) AML12 cells were treated with 100%, 10%, or 0% amino acid-containing media for 24 h. (**A**) Cell viability of AML12 cells for 24 h. The data were min–max normalized. (**B**) Expression levels of *Ddit3*. (**C**,**D**) Expression levels of core clock genes: *Clock* (**C**) and *Per2* (**D**). (**E**–**I**) AML12 cells were treated with either 0.1% of DMSO (control) or 500 nM thapsigargin (Tg) and harvested for qRT-PCR analysis every 6 h. (**E**,**F**) Expression levels of *Atf4* (**E**) and *Ddit3* (**F**) for confirmation of ER stress induction. (**G**–**I**) Expression levels of core clock genes: *Clock* (**G**), *Bmal1* (**H**), and *Per2* (**I**). mRNA expression levels were measured by qRT-PCR analysis, and the data were normalized to the expression of the *Tbp* gene. The data are presented as means ± standard errors for statistical analysis. One-way ANOVA and Tukey’s post hoc test were performed for statistical analysis, and significant differences were indicated by different lowercase letters in (**A**–**D**). *p* < 0.05 was considered statistically significant. Student *t*-tests were used to compare between DMSO and Tg group within each CT in (**E**–**I**) (^#^ *p* < 0.05, ^##^ *p* < 0.01, ^###^ *p* < 0.001). Two-way ANOVA and Tukey’s or Duncan’s post hoc tests were used to determine statistically significant effects of treatment, time, and their interaction (* *p* < 0.05, ** *p* < 0.01, *** *p* < 0.001). The *x*-axis is the treatment time of cells. AA, amino acids; DMSO, dimethyl sulfoxide; CT, circadian time.

To verify whether ER stress had effects on the hepatic circadian rhythm, we treated murine hepatic AML12 cells with 100% (100% AA), 10% (10% AA), or 0% (0% AA) amino acid-containing media for 24 h. Cell viability of AML12 cells treated with 10% and 0% was higher at circadian time (CT) 2 and 4 but lower at CT 16 and 24 compared to the 100% AA group (Figure 5A). Similarly to the in vivo results, ER stress was induced by a low amino acid condition in AML12 cells (Figure 5B). Although the mRNA expression of *Per2* was not different between groups, the expression of *Clock* was altered by a low or no amino acid condition (treatment, *p* = 0.0146) (Figure 5C).

In addition, the AML12 cells were treated with thapsigargin (Tg), a chemical compound that induces ER stress by inhibiting the sarco/endoplasmic reticulum calcium ATPase (SERCA), thereby decreasing calcium ions within the ER [28]. AML12 cells were treated with 500 nM Tg and harvested every 6 h. Tg-induced ER stress was confirmed by the significantly augmented expression levels of *Atf4* and *Ddit3* in the Tg group compared with the dimethyl sulfoxide (DMSO) control group (Figure 5E,F). The expression levels of core clock genes were measured. The mRNA levels of *Clock* (Figure 5G) and *Bmal1* (Figure 5H) were significantly induced by Tg treatment. Although the expression level of *Per2* was not significantly different between the DMSO and Tg groups, significant differences existed depending on the time, and the peak phase was advanced in the Tg group (Figure 5I). Collectively, the ER stress signaling pathway may deliver stress from diet to the hepatic circadian clock.

## 3. Discussion

In this study, we investigated whether dietary protein intake affected the oscillation of the circadian clock, which is an integrative marker for physiological responses, and, if so, screened which molecular pathway was potentially involved.

Previous studies showed that an HPD lowers appetite and weight gain in the elderly [20]. However, intake and BW were not different between the HPD-fed and ND-fed mice in our study. This difference in results might be caused by the high plasticity of protein intake at a young age as well as a difference in species. Solon-Biet et al. showed that LPD-fed mice intake more food and exhibit higher glucose sensitivity than HPD-fed mice [18]. In our results, LPD feeding strongly induced *Fgf21* expression at both ZT 0 and ZT 12. In mice and humans, protein restriction diets or diets reduced in specific amino acids increase the levels of FGF21, which is mainly produced in the liver. Through its interaction with FGF21 receptors in tanycytes, FGF21 is transported to the hypothalamus, and after reaching the brain, it reduces dopamine signaling in brain regions involved in food reward [29]. Our results also showed a higher food and calorie intake in the LPD group compared to the other groups in our study. FGF21 is a stress-response hormone with a number of physiological roles, such as antioxidative stress, anti-inflammation, and lipid reduction [30]. Previous research found that mice administered FGF21 at a dosage of 1 mg/kg/day for 2 weeks exhibited decreased BW, hepatic triglyceride levels, plasma glucose levels, and insulin levels [31]. In our study, we observed that mice in the LPD group had a lower BW, even though they consumed more food. However, its impacts on lipid and glucose metabolism were marginal.

An LPD lowered body weight despite increasing food intake, which has been interpreted as improving metabolic health and anti-obesity; however, it elevated the ER stress response. ER stress is the accumulation of misfolded or unfolded proteins. Various factors may contribute to ER stress, both external, such as nutrient deprivation, hypoxia, and cytotoxic drugs, as well as cell-intrinsic factors [32]. RNA-dependent protein kinase-related endoplasmic reticulum kinase (PERK), inositol-requiring enzyme 1α (IRE1α), and ATF6 are ER transmembrane proteins that sense ER stress [33]. ER contributes to nutrient sensing and metabolic adaptation [34], and the PERK pathway and ATF4 induction are associated with dietary or metabolic stress [35]. For instance, a mouse study showed that a very LPD increased *Atf4* gene expression in the liver depending on protein ratio [26]. Notably, the induction was influenced by the deprivation of dietary protein rather than the levels of dietary fat intake. In our study, the LPD diminished differences in the expression of *Atf4* at ZT 0 and ZT 12, which meant that the oscillation of expression was dampened. Induction of the ER stress response to the LPD was clearer at ZT 12 compared with the ND by showing marginal upregulation of *Atf4* with a *p*-value < 0.1 and significantly upregulated expression of *Ppp1r15a* and *Ddit3*. It was previously revealed that ER stress was induced by tunicamycin-elevated hepatic *Fgf21* and UPR gene expression in male C57BL/6 mice via the eIF2α–ATF4 axis [27]. Fasting and caloric restriction, which are also interpreted as protein restriction conditions, induce an ER stress response even in a FGF21-independent manner [36]. Although the correlation between the ER stress response and FGF21 needs to be clarified, the collective findings imply that it is possible for the ER stress response to act as an intermediary for dietary stress, leading to disruptions in the circadian clock.

The current understanding of the interaction between the ER stress response and circadian clock regulation is limited. Recent studies have provided in vitro evidence suggesting that Tg or tunicamycin-induced ER stress can decrease the amplitude and prolong the period of *Bmal1* oscillation in NIH3T3 cells [37]. Additionally, these treatments have been shown to delay the circadian peak phase of *Bmal1* and *Clock* in U2OS cells, respectively [38]. In a hepatic cell model, we observed circadian clock gene oscillation in AML12 cells treated with DMSO (control). However, when these cells were treated with Tg, ER stress was induced, as shown by the increased *Atf4* and *Ddit3* expression at all time points, and the oscillating expression of core clock genes was disrupted. These findings suggest that an LPD can trigger ER stress, which, in turn, disturbs the normal circadian regulation of clock components. We also investigated whether the effects of AA deprivation on circadian clock regulation were mitigated by employing 4-PBA, an inhibitor of the ER stress pathway (Supplemental Figure S2). *Ddit3* expression indicated an induction of ER stress after 48 h of amino acid deprivation (LAM) compared to the normal amino acid condition (NAM). Furthermore, 4-PBA effectively inhibited the ER stress pathway in a dose-dependent manner. LAM-induced alterations in *Per2* expression were subsequently restored by 4-PBA. These results suggest that LAM-induced ER stress affects circadian clock expression.

In the mouse hypothalamus, the circadian rhythm was not much changed by LPD feeding. However, the period length and/or peak phase of PER2 protein expression were altered in the sub gla, liver, and kidney in accordance with the protein intake level. Furthermore, the changes in the expression of clock-controlled genes induced by an LPD were different between the hypothalamus and liver. Among 351 circadian genes, only 10 circadian clock genes in the hypothalamus were significantly changed by an LPD, while 39 genes were changed in the liver. Previous research revealed that diet-induced modulation of circadian regulation showed tissue specificity [39,40]. Not only tissue specificity but also the independence of the central clock can be a reason [41].

There are several limitations to this study. Firstly, while we observed that an LPD can potentially affect the circadian rhythm by inducing ER stress, the direction of change in the circadian rhythm was not clear throughout the study. Additionally, we were unable to identify the specific pathway through which ER stress signals are transmitted to the peripheral clock, and there is even a possibility that the effects of an LPD on the peripheral clock are due to another pathway, such as GCN2 activation, rather than ER stress. Therefore, further research is needed to clarify the correlation between the low-protein condition, ER stress response, and circadian rhythm regulation, as well as to determine the specific mechanism involved. This includes investigating ER stress signaling pathways such as PERK, IRE1α, and ATF6. Secondly, our analysis was limited to a small number of metabolic biomarkers and two time points, ZT 0 and ZT 12, to assess circadian changes. Because the peak time and oscillation phase for each circadian component can vary, continuous monitoring is required to investigate these factors more comprehensively. Lastly, further analysis with various protein distributions and feeding periods is necessary to draw more definitive conclusions.

## 4. Materials and Methods

### 4.1. Secondary Analysis of Transcriptome Data Using Gene Expression Omnibus (GEO) Datasets

A secondary analysis of the hypothalamic transcriptome was performed using publicly available RNA-sequencing data: GEO accession number GSE158215 [26]. The data included transcriptomes from murine hypothalamus samples of the LPD, LP/HFD, and ND groups. The data were quality-controlled using FastQC v0.11.9 software (http://www.bioinformatics.babraham.ac.uk/projects/fastqc/, accessed on 19 April 2021) [42]. The reads were mapped to the mouse reference genome GRCm38 (Mus_musculus.GRCm38.dna.primary_assembly.fa) from Ensembl using the Spliced Transcripts Alignment to a Reference (STAR) program [43]. An annotation file (Mus_musculus.GRCm38.102.gtf) was obtained from the Ensembl website (http://www.ensembl.org, accessed on 19 April 2021). The counting of reads was conducted with FeatureCounts [44], followed by the analysis of DEGs using the DEseq2 package in R version 4.1.1. DEGs were identified based on a *p*-value cut-off of 0.05. We used GO terms associated with the circadian rhythm (GO:0007623) to investigate the effects of protein intake on circadian gene expression. Pathway analysis was conducted using the Kyoto Encyclopedia of Genes and Genomes (KEGG) database. The Heatmapper online application (http://heatmapper.ca, accessed on 19 April 2021; Wishart Research Group, University of Alberta, Edmonton, AB, Canada) was used for expression heatmaps.

### 4.2. mPer2::Luc Knock-in Mice Model

Male *mPer2::Luc* knock-in mice (hetero-type; JAX stock #006852) [45] with a pure C57BL/6N background were originally acquired from the Jackson Laboratory (Bar Harbor, ME, USA) and further derived at the Laboratory Animal Resource Center (LARC), Korea Research Institute of Bioscience and Biotechnology (KRIBB, Daejeon, Republic of Korea). Through breeding with wild-type C57BL/6N female mice, male *mPer2::Luc* knock-in mice (hetero-type) were obtained and used for the experiment. Mice were maintained at 21–22 °C under a 12 h:12 h light:dark cycle with lights on at 7 am. The average BW of mice measured at 5 weeks was not significantly different between the three diet groups. They were fed with an ND (AIN-93G: 20% protein, 16% fat, 64% carbohydrate), HPD (33% protein, 20% fat, 47% carbohydrate), or LPD (5% protein, 20% fat, 75% carbohydrate) ad libitum for 6 weeks, starting at 5 weeks of age. Details of the diet compositions are presented in Supplementary Table S1.

BW and food intake were measured every week, and an intraperitoneal (I.P.) glucose tolerance test (IPGTT), in vivo monitoring of PER2, and tissue collection were conducted 6 weeks after starting the dietary intervention. The tissue weight was normalized to the final BW. The IPGTT was conducted after fasting mice for 16 h (ZT 13–ZT 29). Glucose solution (1 g/kg of BW) was injected I.P. Blood collected from the tail vein at 0, 15, 30, 60, 90, and 120 min after glucose injection (I.P.) was analyzed for glucose concentration using the Accu-Chek Active glucometer (Roche Diagnostics Korea, Seoul, Republic of Korea).

All the animal studies were carried out in accordance with the guidelines of the Institutional Animal Care and Use Committee (IACUC) of Ewha Womans University (IACUC 20-055, 21-032).

### 4.3. In Vivo Bioluminescence Measurement

In vivo monitoring of PER2::LUC bioluminescence in the peripheral tissues of *mPer2::Luc* knock-in mice was performed using an imaging system (IVIS Lumina Series III, PerkinElmer, Waltham, MA, USA). Mice were anesthetized using a gas anesthesia system (XGI-8, PerkinElmer). Under anesthesia, mice were shaved with clippers, and then D-luciferin potassium salt (Promega, Madison, WI, USA) was injected at a dose of 15 mg/kg in Dulbecco’s phosphate-buffered saline (DPBS, Welgene, Daegu, Republic of Korea; LB001-02). Images were captured six times per day (ZT: 0, 4, 8, 12, 16, and 20) in the dorsal-up position for the kidney and the ventral-up position for the liver and sub gla. Images were obtained with a 1 min exposure time at 8 min after the I.P. injection. The region-of-interest (ROI) was set up with the same area and location in each organ data analysis using Living Image 4.5 software (PerkinElmer).

### 4.4. Cell Culture

The murine hepatocyte cell line AML12 was gratefully provided by Dr. Sujin Yang’s laboratory (Seoul Women’s University, Seoul, Republic of Korea), who originally purchased it from the American Type Culture Collection (ATCC, Manassas, VA, USA). The AML12 cells were cultured in growth media containing DMEM/F-12 (Welgene; LM002-08) with 10% fetal bovine serum (Corning Inc., Corning, NY, USA; 35-015-CV) and 1% penicillin-streptomycin (Gibco, Gaithersburg, MD, USA; 15140-122). Cells were seeded in 6-well plates for media with altered amino acid composition or Tg (Sigma-Aldrich, St. Louisan, MO, USA; T9033) treatment, then, after 24 h, synchronized with 50% horse serum for 2 h. The medium with altered amino acid composition included 100%, 10%, or 0% of amino acids, and the amino acid concentration of 100% amino acid media was referenced to the composition of DMEM/F-12. Tg was diluted to 500 mM in DMSO (Sigma-Aldrich, D2650) and further diluted in culture medium to a final concentration of 500 nM. Cells were then treated with this solution and harvested every 6 h for total RNA isolation.

### 4.5. Quantitative Real-Time PCR (q-PCR)

Total RNA was extracted from AML12 cells using TRIzol reagent (Ambion, Austin, TX, USA; 15596018) or the RNeasy Kit (QIAGEN, Hilden, Germany) according to the manufacturer’s protocol. Total RNA from about 15 mg of liver tissue was extracted using the RNeasy Kit (QIAGEN) according to the manufacturer’s protocol. The q-PCR was conducted with cDNA, transcript-specific primers, and SYBR Green PCR Master Mix (QIAGEN; 204074) using a Rotor-Gene Q system (QIAGEN). Each sample was measured in duplicate, and relative expression levels were calculated by the 2^−ΔΔ*C*^*^t^* method and normalized to the expression of TATA-box binding protein (*Tbp*). Primer sequences are presented in Supplementary Table S2.

### 4.6. Statistical Analysis

The means of the two groups were statistically analyzed by a Student’s two-tailed *t*-test with equal variances using Microsoft Excel (ver. 2016; Microsoft, Redmond, WA, USA). The means of more than two groups were statistically analyzed by one-way ANOVA with Tukey’s or Duncan’s post hoc test. For the analysis of data from more than one variable, a two-way ANOVA and Tukey’s or Duncan’s post hoc test were performed. To test the tendency of gene expression depending on dietary protein intake, we used linear regression analysis. To test the rhythmicity of bioluminescence data, we used the JTK_CYCLEv3.1 non-parametric test [46] in R Studio, using a window of 20–28 h to find circadian oscillations. Values are expressed as the means ± standard errors. A *p*-value < 0.05 denotes statistical significance.

## 5. Conclusions

Although the LPD did not influence the hypothalamic central clock, it altered peripheral clock regulation. The LPD significantly changed the circadian oscillation of PER2 in the sub gla, liver, and kidney according to in vivo PER2 monitoring, whereas the HPD group showed similar phenotypic and molecular parameters to the ND group. Dietary protein intake, in particular an LPD, modified the oscillating expression of ER stress-response genes, to which dietary stress-induced alteration of circadian clock oscillation was attributed. Further studies are required to decipher the correlation between ER stress response and circadian rhythm regulation, as well as to identify specific ER stress signaling pathways. Additionally, more research is necessary to fully understand the effects of dietary protein intake using a wider range of biomarkers and various levels of dietary protein intake.

## Data Availability

Data generated or analyzed during this study are available upon request.

## Supplementary Materials

The following supporting information can be downloaded at: https://www.mdpi.com/article/10.3390/ijms25137373/s1.

## References

[1] Bass J., Takahashi J.S. (2010). Circadian integration of metabolism and energetics. Science.

[2] Stenvers D.J., Scheer F.A., Schrauwen P., la Fleur S.E., Kalsbeek A. (2019). Circadian clocks and insulin resistance. Nat. Rev. Endocrinol..

[3] Stenvers D.J., Jongejan A., Atiqi S., Vreijling J.P., Limonard E.J., Endert E., Baas F., Moerland P.D., Fliers E., Kalsbeek A. (2019). Diurnal rhythms in the white adipose tissue transcriptome are disturbed in obese individuals with type 2 diabetes compared with lean control individuals. Diabetologia.

[4] Sato S., Solanas G., Peixoto F.O., Bee L., Symeonidi A., Schmidt M.S., Brenner C., Masri S., Benitah S.A., Sassone-Corsi P. (2017). Circadian reprogramming in the liver identifies metabolic pathways of aging. Cell.

[5] Hood S., Amir S. (2017). The aging clock: Circadian rhythms and later life. J. Clin. Investig..

[6] Patel S.A., Velingkaar N., Makwana K., Chaudhari A., Kondratov R. (2016). Calorie restriction regulates circadian clock gene expression through BMAL1 dependent and independent mechanisms. Sci. Rep..

[7] Kinouchi K., Magnan C., Ceglia N., Liu Y., Cervantes M., Pastore N., Huynh T., Ballabio A., Baldi P., Masri S. (2018). Fasting imparts a switch to alternative daily pathways in liver and muscle. Cell Rep..

[8] Froy O., Chapnik N., Miskin R. (2009). Effect of intermittent fasting on circadian rhythms in mice depends on feeding time. Mech. Ageing Dev..

[9] Sherman H., Genzer Y., Cohen R., Chapnik N., Madar Z., Froy O. (2012). Timed high-fat diet resets circadian metabolism and prevents obesity. FASEB J..

[10] Hatori M., Vollmers C., Zarrinpar A., DiTacchio L., Bushong E.A., Gill S., Leblanc M., Chaix A., Joens M., Fitzpatrick J.A. (2012). Time-restricted feeding without reducing caloric intake prevents metabolic diseases in mice fed a high-fat diet. Cell Metab..

[11] Adamovich Y., Rousso-Noori L., Zwighaft Z., Neufeld-Cohen A., Golik M., Kraut-Cohen J., Wang M., Han X., Asher G. (2014). Circadian clocks and feeding time regulate the oscillations and levels of hepatic triglycerides. Cell Metab..

[12] Mitchell S.J., Bernier M., Mattison J.A., Aon M.A., Kaiser T.A., Anson R.M., Ikeno Y., Anderson R.M., Ingram D.K., de Cabo R. (2019). Daily fasting improves health and survival in male mice independent of diet composition and calories. Cell Metab..

[13] Kuroda H., Tahara Y., Saito K., Ohnishi N., Kubo Y., Seo Y., Otsuka M., Fuse Y., Ohura Y., Hirao A. (2012). Meal frequency patterns determine the phase of mouse peripheral circadian clocks. Sci. Rep..

[14] Shon J., Han Y., Park Y.J. (2022). Effects of Dietary Fat to Carbohydrate Ratio on Obesity Risk Depending on Genotypes of Circadian Genes. Nutrients.

[15] Vollmers C., Gill S., DiTacchio L., Pulivarthy S.R., Le H.D., Panda S. (2009). Time of feeding and the intrinsic circadian clock drive rhythms in hepatic gene expression. Proc. Natl. Acad. Sci. USA.

[16] Ribas-Latre A., Eckel-Mahan K. (2016). Interdependence of nutrient metabolism and the circadian clock system: Importance for metabolic health. Mol. Metab..

[17] Eckel-Mahan K.L., Patel V.R., De Mateo S., Orozco-Solis R., Ceglia N.J., Sahar S., Dilag-Penilla S.A., Dyar K.A., Baldi P., Sassone-Corsi P. (2013). Reprogramming of the circadian clock by nutritional challenge. Cell.

[18] Solon-Biet S.M., McMahon A.C., Ballard J.W.O., Ruohonen K., Wu L.E., Cogger V.C., Warren A., Huang X., Pichaud N., Melvin R.G. (2014). The ratio of macronutrients, not caloric intake, dictates cardiometabolic health, aging, and longevity in ad libitum-fed mice. Cell Metab..

[19] Appel L.J., Sacks F.M., Carey V.J., Obarzanek E., Swain J.F., Miller E.R., Conlin P.R., Erlinger T.P., Rosner B.A., Laranjo N.M. (2005). Effects of protein, monounsaturated fat, and carbohydrate intake on blood pressure and serum lipids: Results of the OmniHeart randomized trial. JAMA.

[20] Pasiakos S.M., McLellan T.M., Lieberman H.R. (2015). The effects of protein supplements on muscle mass, strength, and aerobic and anaerobic power in healthy adults: A systematic review. Sports Med..

[21] Analysts G.I. (2012). Protein Ingredients: A Global Strategic Business Report.

[22] Park Y.J., Chung S., Hwang J.-T., Shon J., Kim E. (2022). A review of recent evidence of dietary protein intake and health. Nutr. Res. Pract..

[23] Solon-Biet S.M., Mitchell S.J., Coogan S.C., Cogger V.C., Gokarn R., McMahon A.C., Raubenheimer D., de Cabo R., Simpson S.J., Le Couteur D.G. (2015). Dietary protein to carbohydrate ratio and caloric restriction: Comparing metabolic outcomes in mice. Cell Rep..

[24] Levine M.E., Suarez J.A., Brandhorst S., Balasubramanian P., Cheng C.-W., Madia F., Fontana L., Mirisola M.G., Guevara-Aguirre J., Wan J. (2014). Low protein intake is associated with a major reduction in IGF-1, cancer, and overall mortality in the 65 and younger but not older population. Cell Metab..

[25] Simpson S.J., Le Couteur D.G., James D.E., George J., Gunton J.E., Solon-Biet S.M., Raubenheimer D. (2017). The geometric framework for nutrition as a tool in precision medicine. Nutr. Healthy Aging.

[26] Wu Y., Li B., Li L., Mitchell S.E., Green C.L., D’Agostino G., Wang G., Wang L., Li M., Li J. (2021). Very-low-protein diets lead to reduced food intake and weight loss, linked to inhibition of hypothalamic mTOR signaling, in mice. Cell Metab..

[27] Kim S.H., Kim K.H., Kim H.-K., Kim M.-J., Back S.H., Konishi M., Itoh N., Lee M.-S. (2015). Fibroblast growth factor 21 participates in adaptation to endoplasmic reticulum stress and attenuates obesity-induced hepatic metabolic stress. Diabetologia.

[28] Treiman M., Caspersen C., Christensen S.B. (1998). A tool coming of age: Thapsigargin as an inhibitor of sarco-endoplasmic reticulum Ca2+-ATPases. Trends Pharmacol. Sci..

[29] Hill C.M., Laeger T., Dehner M., Albarado D.C., Clarke B., Wanders D., Burke S.J., Collier J.J., Qualls-Creekmore E., Solon-Biet S.M. (2019). FGF21 signals protein status to the brain and adaptively regulates food choice and metabolism. Cell Rep..

[30] Xiao M., Tang Y., Wang S., Wang J., Guo Y., Zhang J., Gu J. (2021). The Role of Fibroblast Growth Factor 21 in Diabetic Cardiovascular Complications and Related Epigenetic Mechanisms. Front. Endocrinol..

[31] BonDurant L.D., Ameka M., Naber M.C., Markan K.R., Idiga S.O., Acevedo M.R., Walsh S.A., Ornitz D.M., Potthoff M.J. (2017). FGF21 regulates metabolism through adipose-dependent and-independent mechanisms. Cell Metab..

[32] Chen X., Cubillos-Ruiz J.R. (2021). Endoplasmic reticulum stress signals in the tumour and its microenvironment. Nat. Rev. Cancer.

[33] Wang M., Wey S., Zhang Y., Ye R., Lee A.S. (2009). Role of the unfolded protein response regulator GRP78/BiP in development, cancer, and neurological disorders. Antioxid. Redox Signal..

[34] Lemmer I.L., Willemsen N., Hilal N., Bartelt A. (2021). A guide to understanding endoplasmic reticulum stress in metabolic disorders. Mol. Metab..

[35] Fernandes-da-Silva A., Miranda C.S., Santana-Oliveira D.A., Oliveira-Cordeiro B., Rangel-Azevedo C., Silva-Veiga F.M., Martins F.F., Souza-Mello V. (2021). Endoplasmic reticulum stress as the basis of obesity and metabolic diseases: Focus on adipose tissue, liver, and pancreas. Eur. J. Nutr..

[36] Capelo-Diz A., Lachiondo-Ortega S., Fernández-Ramos D., Cañas-Martín J., Goikoetxea-Usandizaga N., Serrano-Maciá M., González-Rellan M.J., Mosca L., Blazquez-Vicens J., Tinahones-Ruano A. (2023). Hepatic levels of S-adenosylmethionine regulate the adaptive response to fasting. Cell Metab..

[37] Gao L., Chen H.T., Li C.M., Xiao Y.Y., Yang D., Zhang M.H., Zhou D., Liu W., Wang A.H., Jin Y.P. (2019). ER stress activation impairs the expression of circadian clock and clock-controlled genes in NIH3T3 cells via an ATF4-dependent mechanism. Cell Signal.

[38] Bu Y.W., Yoshida A., Chitnis N., Altman B.J., Tameire F., Oran A., Gennaro V., Armeson K.E., McMahon S.B., Wertheim G.B. (2018). A PERK-miR-211 axis suppresses circadian regulators and protein synthesis to promote cancer cell survival. Nat. Cell Biol..

[39] Tognini P., Murakami M., Liu Y., Eckel-Mahan K.L., Newman J.C., Verdin E., Baldi P., Sassone-Corsi P. (2017). Distinct Circadian Signatures in Liver and Gut Clocks Revealed by Ketogenic Diet. Cell Metab..

[40] Dyar K.A., Lutter D., Artati A., Ceglia N.J., Liu Y., Armenta D., Jastroch M., Schneider S., de Mateo S., Cervantes M. (2018). Atlas of circadian metabolism reveals system-wide coordination and communication between clocks. Cell.

[41] Finger A.-M., Kramer A. (2021). Peripheral clocks tick independently of their master. Genes Dev..

[42] Andrews S. (2010). Babraham Bioinformatics-FastQC a Quality Control Tool for High Throughput Sequence Data. https://www.bioinformatics.babraham.ac.uk/projects/fastqc.

[43] Dobin A., Davis C.A., Schlesinger F., Drenkow J., Zaleski C., Jha S., Batut P., Chaisson M., Gingeras T.R. (2013). STAR: Ultrafast universal RNA-seq aligner. Bioinformatics.

[44] Liao Y., Smyth G.K., Shi W. (2014). featureCounts: An efficient general purpose program for assigning sequence reads to genomic features. Bioinformatics.

[45] Yoo S.-H., Yamazaki S., Lowrey P.L., Shimomura K., Ko C.H., Buhr E.D., Siepka S.M., Hong H.-K., Oh W.J., Yoo O.J. (2004). PERIOD2:: LUCIFERASE real-time reporting of circadian dynamics reveals persistent circadian oscillations in mouse peripheral tissues. Proc. Natl. Acad. Sci. USA.

[46] Hughes M.E., Hogenesch J.B., Kornacker K. (2010). JTK_CYCLE: An efficient nonparametric algorithm for detecting rhythmic components in genome-scale data sets. J. Biol. Rhythm..

