# Supplementary material for: Effects of Dietary Protein Intake Levels on Peripheral Circadian Rhythm in Mice
Source: Int J Mol Sci. 2024 Jul 5;25(13):7373. doi: 10.3390/ijms25137373 (PMC11242084; doi:10.3390/ijms25137373)
Supplement: Supplementary file 1 [file ijms-25-07373-s001.zip › ijms-3082387-supplementary.pdf]

Supplemental Table S1. Compositions of diets and calorie % from total energy

Ingredients (g/kg)	LPD	ND	HPD
Casein	50.03	200.08	330.63
L-Cystine	0.75	3.00	4.51
Corn Starch	600.39	397.16	315.60
Maltodextrin	100.07	132.05	100.19
Sucrose	51.03	100.04	51.10
Cellulose	50.03	50.02	50.10
Soybean oil	90.06	70.03	90.17
Lard	0.00	0.00	0.00
Kcal %			
Protein	5.0	20.3	33.0
Carbohydrate	75.0	63.9	47.0
Fat	20.0	15.8	20.0
Energy (kcal/gm)	4.1	4.0	4.1

Supplemental Table S2. Primer sequences for qRT-PCRs

Gene name	Gene accession number	Species		Primer sequence (5'-3')
Tbp	NM_013684.3	Mouse	Forward	GACCAGAACAACAGCCTTCC
			Reverse	GAGTAAGTCCTGTGCCGTAAG
Clock	NM_001289826.1	Mouse	Forward	GCAGCTTCCTTCAGTTCAGC
			Reverse	GAAAGGCAGCAGAGAGGATG
Bmal1	NM_007489.4	Mouse	Forward	CCCGCTGAACATCACAAGTA
			Reverse	TGAGCCTGCCCTGGTAATAG
Per2	NM_011066.3	Mouse	Forward	GAAGGAGGACCAGGAGAAGC
			Reverse	TGCCTTTCTCCTCACTCTCG
Per3	NM_011067.3	Mouse	Forward	TCATGACATACCAGGTGCCC
			Reverse	CTGTGTGGCTGTGGATCCA
Cry1	NM_007771.3	Mouse	Forward	CAGACTCACTCACTCAAGCAAGG
			Reverse	TCAGTTACTGCTCTGCCGCTGGAC
Dbp	NM_016974.3	Mouse	Forward	AATGACCTTTGAACCTGATCCCGCT
			Reverse	GCTCCAGTACTTCTCATCCTTCTGT
Nampt	NM_021524.2	Mouse	Forward	GGCGCTTTGCTACAGAAGTT
			Reverse	TTTGTTGGGATCAGCAACTG
Fgf21	NM_020013.4	Mouse	Forward	CAAATCCTGGGTGTCAAAGC
			Reverse	ATGGGCCTCAGACTGGTACA
Pck1	NM_011044.2	Mouse	Forward	ATGAAGTTTGATGCCCAAGG
			Reverse	CGTTGGTGAAGATGGTGTTTT
Gcg	NM_008100.4	Mouse	Forward	CCAGGTGGTCATGTCTTCTG
			Reverse	GCATTGTGTAACCCAACGATT
Irs1	NM_010570.4	Mouse	Forward	AAGACGTGAGGTCCTGGTTG
			Reverse	GCCAGAGGATCGTCAATAGC
Gck	NM_001287386.1	Mouse	Forward	TGGTGGATGAGAGCTCAGTG
			Reverse	CCTCTCCGTGGAACAGAAGA
G6pdx	NM_008062.3	Mouse	Forward	GGTGTAACTTCCAGGGCTCA
			Reverse	CACCACTGCTGCACAAGATT
Fasn	NM_007988.3	Mouse	Forward	GTTGGGGGTGTCTTCAACCT
			Reverse	CAGCTCAGGGCAGGCTTC
Acc1	NM_133360.3	Mouse	Forward	AGTGGCTGGAGAAGCAACTG
			Reverse	GAGATGTGCTGGGTCATGTG
Atgl	NM_001163689.1	Mouse	Forward	TACTGTGGCCTCATTCCTCC
			Reverse	GGGGACACTGTGATGGTATTC
Pgc1a	NM_008904.3	Mouse	Forward	TTACACCTGTGACGCTTTCG
			Reverse	GTGGAAGCAGGGTCAAAATC
Cpt1a	NM_013495.2	Mouse	Forward	TGCCTCTATGTGGTGTCCAA
			Reverse	AAACAGTTCCACCTGCTGCT
Pparg	NM_001127330.3	Mouse	Forward	GCCCTTTGGTGACTTTATGG
			Reverse	CATCTTCTGGAGCACCTTGG
Atf4	NM_009716.3	Mouse	Forward	CCACCATGGCGTATTAGAGG
			Reverse	CTTCGCTGTTCAGGAAGCTC
Atf6	NM_001081304.1	Mouse	Forward	TACCACCCACAACAAGACCA
			Reverse	TGATGATCCCGGAGATAAGG
Ddit3	NM_007837.4	Mouse	Forward	GGAGGTCCTGTCCTCAGATG
			Reverse	GGACGCAGGGTCAAGAGTAG
Ppp1r15a	NM_008654.2	Mouse	Forward	CGGCTCAGATTGTTCAAAGC
			Reverse	CAGCAAGGAAATGGACTGTG

Supplemental Figure S1. Results of secondary analysis of public microarray data indicating altered UPR response and expression of circadian genes in mouse liver in high fat diet (HFD) group.

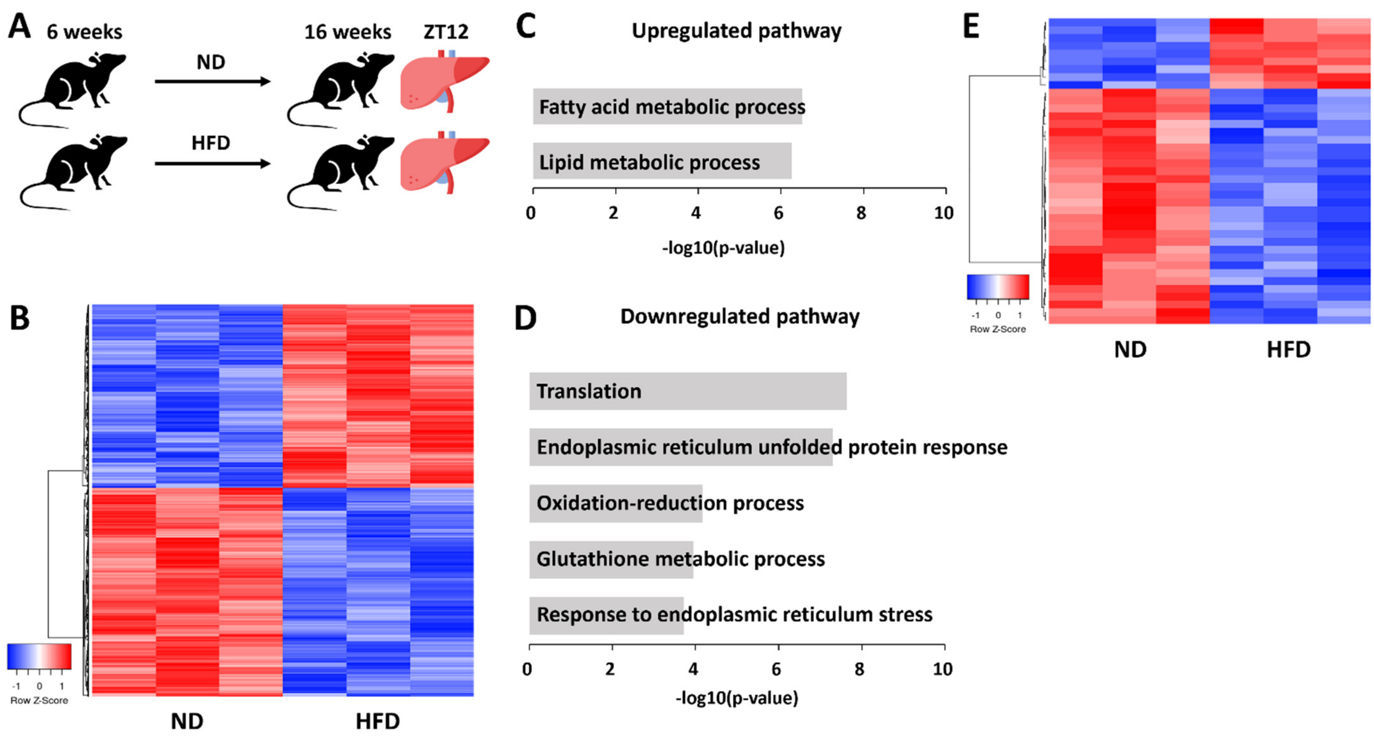

(A) Experimental design of GSE52333. (B) Heatmap presenting expression of DEGs ($p < 0.01$) in HFD group versus normal diet (ND) group. (C) Upregulated pathway in results of gene ontology analysis with p-value < 0.01 and FDR < 0.05. (D) Downregulated pathway in results of gene ontology analysis with p-value < 0.01 and FDR < 0.05. (E) Heatmap presenting genes involved in Gene Ontology term “Circadian rhythm” (GO:0007623) among DEGs. Among 691 DEGs, 39 circadian genes were differentially expressed with significance ($p < 0.01$). In (B) and (E), each column represents a sample, and each row represents a gene. Red and blue color indicates up-regulated genes and down-regulated genes, respectively.

Supplemental Figure S2. Impacts of amino acids deprivation and ER stress inhibition with 4-PBA on *Ddit3*, *Clock*, and *Per2* expression in AML12 cells

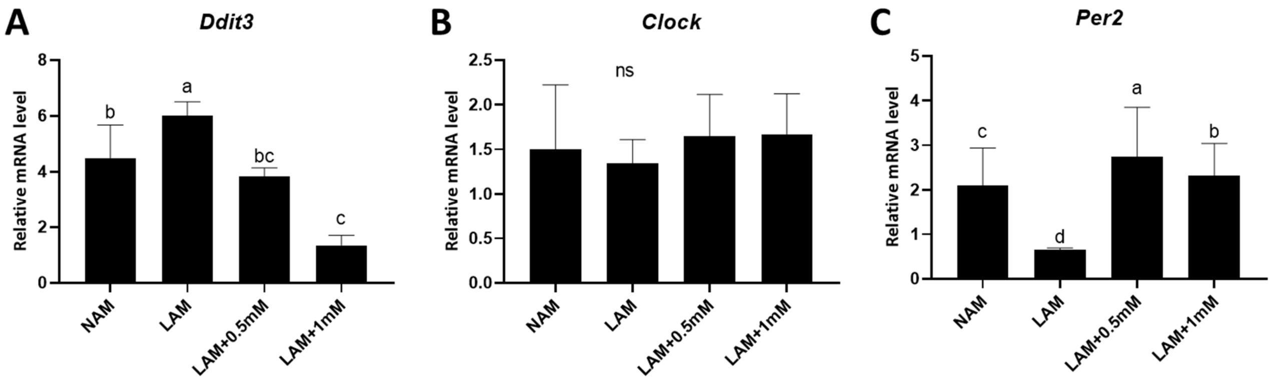

AML12 cells were maintained in normal amino acids media (NAM, 100% amino acids) or low amino acids media (LAM, 25% amino acids) with or without 4-PBA, which is blocker of ER stress. Treatment with 4-PBA was conducted at a final concentration of 0.5 or 1 mM. After 48 hours, cells were harvested for the measurement of *Ddit3* (A), *Clock* (B), and *Per2* (C) expression. N=2 biological replicates. Data are presented as the means ± standard errors. A mixed model with least squares and Tukey's *post hoc* test were used for statistical analysis, indicated by different letters. A p-value < 0.05 was considered statistically significant.
